# Hebbian cross-correlation learning emerges as spike timing dependent plasticity

**DOI:** 10.1186/1471-2202-12-S1-P355

**Published:** 2011-07-18

**Authors:** Tjeerd olde Scheper, Rhiannon Meredith, Huibert Mansvelder, Jaap van Pelt, Arjen van Ooyen

**Affiliations:** 1 Department of Integrative Neurophysiology, Center for Neurogenomics and Cognitive Research (CNCR), Vrije Universiteit Amsterdam, De Boelelaan 1085, 1081 HV, Amsterdam, The Netherlands; 2 Department of Computing, Oxford Brookes University, Wheatley Campus, Oxford, OX33 1HX, UK

## 

In Donald O. Hebb's oft quoted thesis on synaptic plasticity, the change in efficacy in proportion to the degree of correlation between pre- and post-synaptic activity is expressed explicitly. The Hebbian learning rule has been demonstrated in simulations to be reliable and effective and appears to have a solid foundation in biology on the basis of experimental results. However, beyond binary simulation models of the Spike Timing Dependent Plasticity (STDP) rule, it has not been demonstrated that the causal correlation property of synaptic plasticity is as valid and as effective as always has been assumed.

To clarify the exact nature of learning by means of spike timing dependent plasticity, a dynamic model has been developed based on the cross-correlation between pre- and post-synaptic activity as expressed by a dynamic activity measure. The components that form the model are centered around the following guiding principles. Firstly, the cross-correlation between local synaptic pre- and post-synaptic activity, as induced by the synapse itself in the post-synaptic cell, determines the strength of the potential for synaptic depression. Even though this may appear to be counter-intuitive, it reflects the depression due to pre-synaptic activity if little or no subsequent post-synaptic activity is present. The second component is the post-synaptic activity induced by the synapse locally. This represents the local response to synaptic input. The third component is the cross-correlation of the post-synaptic activity induced by the synapse with other post-synaptic activity contributing factors such as an action potential and other synapses. These three components form the autonomous learning rule from which Spike Timing Dependent Plasticity learning emerges. Due to the dynamic nature of the autonomous learning rule, it responds in a simple feed-forward manner to the synaptic input in combination to the localised post-synaptic activity. This precludes the need to perform spike matching and post-processing of the simulation and is more biologically relevant.

The relation between the local dynamics of a single synapse and the localised dynamics due to post-synaptic activity becomes apparent by different emerging learning rules. The presence of action potentials and synaptic inhibition can change the shape of the STDP learning rule even to the extent that a Hebbian learning rule may become anti-Hebbian and vice versa. The synapse can respond to external input as well as compete with other synapses and tune itself to the local dendritic activity and the global neuronal activity.

Synaptic adaptation due to the presence of nearby synapses and global activity has previously not been extensively studied. This work shows that synapses are not mere slaves to the input but perform more complex computations by combining the input with the local post-synaptic activity as well as the global dynamics due to other synapses and action potentials.

